# Supplementary material for: A meritocratic network formation model for the rise of social media influencers
Source: Nat Commun. 2021 Nov 30;12:6865. doi: 10.1038/s41467-021-27089-8 (PMC8633025; doi:10.1038/s41467-021-27089-8)
Supplement: Supplementary file 1 — Supplementary Information [file 41467_2021_27089_MOESM1_ESM.pdf]

Supplementary Information for “A meritocratic network formation model for the rise of social media influencers”

Nicolò Pagan et al.

Social Computing Group, University of Zürich, Andreasstrasse 15 8050 Zürich, Switzerland.

nicolo.pagan@uzh.ch

Supplementary Information for “A meritocratic network formation model for the rise of social media influencers”

Nicolò Pagan et al.

Social Computing Group, University of Zürich, Andreasstrasse 15 8050 Zürich, Switzerland.

nicolo.pagan@uzh.ch

The Supplementary Information is organized as follows: in Supplementary Note 1, we present the proof of the theorems on the in-degree and out-degree probability density functions related to our quality-based model. Additionally, we present the results of the model in the special case of a bipartite-like network. In Supplementary Note 2, we complement the theoretical analysis with some numerical extensions. In particular, we analyze the effect of an in-degree based preferential attachment meeting process. and of multiple nodes with the same quality. In Supplementary Note 3 we describe the Twitch data-sets collection method and in Supplementary Note 4 we complement the analysis presented in the manuscript.

Supplementary Note 1. *Analytical Proofs*

Theorem (In-degree distribution)**.** *The probability that node i is followed by node $j \neq i$ after $t > 0$ time-steps is:*

$$\mathsf{P}\left[a_{ji}(t)=1\right] = \begin{cases} \bar{p}_i(t) := \frac{1}{i-1}\left(1-\left(\frac{N-i}{N-1}\right)^t\right), & \text{if } j<i, \\ \underline{p}_i(t) := \frac{1}{i}\left(1-\left(\frac{N-i-1}{N-1}\right)^t\right), & \text{if } j>i. \end{cases} \quad (1)$$

Moreover, the probability of node i having in-degree $d_i^{\text{in}}(t) = d \in [0, N-1]$ after t time-steps is given by

$$\mathsf{P}\left[d_i^{\text{in}}(t)=d\right] = \quad (2)$$
$$\sum_{k=0}^{d} \binom{i-1}{k} \bar{p}_i^k \left(1-\bar{p}_i\right)^{i-1-k} \binom{N-i}{d-k} \underline{p}_i^{d-k} \left(1-\underline{p}_i\right)^{N-i-(d-k)},$$

where we omitted the time-step dependency on $\bar{p}_i$ and $\underline{p}_i$. Finally, the expected in-degree of node i after t time-steps reads as:

$$\mathbb{E}\left[d_i^{\text{in}}(t)\right] = \frac{N}{i} - \left(\left(\frac{N-i}{N-1}\right)^t + \frac{N-i}{i}\left(\frac{N-i-1}{N-1}\right)^t\right). \quad (3)$$

Proof. First, we consider $j < i$. The event of j linking to i at time-step t only depends on the previous choices of j. If j meets i when the set of j's followees is empty (first choice), then j will certainly follow i. This happens with probability $1/(N-1)$. Otherwise, j might follow i at her k^{th} choice if and only if q_i is higher than every other q_l in the current set of j's followees. Since there are $N-i$ nodes with $q_l < q_i$, then the probability of j following i at her k^{th} choice is equal to

$$\mathsf{P}\left[a_{ji}(k)=1\right] = \left(\frac{N-i}{N-1}\right)^{k-1}\frac{1}{N-1}.$$

Then, the probability of $j < i$ following i after t time-steps is equivalent to the sum of the probability of j following i at her first, second, ..., $k^{th} \le t^{th}$ choice, i.e.,

$$\begin{aligned} \mathsf{P}\left[a_{ji}(t)=1\right] &= \sum_{k=1}^{t}\left(\frac{N-i}{N-1}\right)^{k-1}\frac{1}{N-1} = \\ &= \frac{\left(\frac{N-i}{N-1}\right)^t - 1}{\frac{N-i}{N-1}-1}\frac{1}{N-1} = \\ &= \frac{1}{i-1}\left(1-\left(\frac{N-i}{N-1}\right)^t\right) =: \bar{p}_i(t). \end{aligned}$$

On the other hand, if $j > i$ then there are only $N-i-1$ nodes $l \neq j$ such that $q_l < q_i$. Thus, the probability of $j > i$ following i after t time-steps reads as:

$$\begin{aligned} \mathsf{P}\left[a_{ji}(t)=1\right] &= \sum_{k=1}^{t}\left(\frac{N-i-1}{N-1}\right)^{k-1}\frac{1}{N-1} = \\ &= \frac{1}{i}\left(1-\left(\frac{N-i-1}{N-1}\right)^t\right) =: \underline{p}_i(t). \end{aligned}$$

This concludes the first part of the proof. Note that $\bar{p}_i(t) = \underline{p}_{i-1}(t)$ for all $i = 2, \ldots, N$.

For the second part of the proof, note that the probability distribution of the in-degree of the node i is a Poisson binomial distribution of $N-1$ independent experiments, i.e., the sum of $N-1$ independent Bernoulli trials which are not necessarily identically distributed. The jth experiment, with $j = 1, \ldots, i-1, i+1, \ldots, N$, is related to the link $a_{ji}(t)$, which is a Bernoulli random variable with probability distribution described by (1). Note that $a_{ji}(t)$

is independent from $a_{ki}(t)$, for all $k \neq j$. Note also that there are $i-1$ nodes such that $j < i$, and $N-i$ nodes such that $j > i$. Thus, the probability distribution of the in-degree of node i can be described as

$$\mathsf{P}\left[d_i^{\text{in}}(t) = d\right] = \sum_{k=0}^{d} \binom{i-1}{k} \bar{p}_i^k \left(1-\bar{p}_i\right)^{i-1-k} \binom{N-i}{d-k} \underline{p}_i^{d-k} \left(1-\underline{p}_i\right)^{N-i-(d-k)},$$

where we omitted the argument t in $\bar{p}_i$ and $\underline{p}_i$.

Moreover, for a Poisson binomial distribution of n independent experiments, each with probability p_l of success, the expectation reads as $\sum_l p_l$. In this case,

$$\begin{aligned}\mathbb{E}\left[d_i^{\text{in}}(t)\right] &= \sum_{j<i} \bar{p}_i(t) + \sum_{j>i} \underline{p}_i(t) = \\ &= (i-1)\,\bar{p}_i(t) + (N-i)\,\underline{p}_i(t) = \\ &= \frac{N}{i} - \left(\left(\frac{N-i}{N-1}\right)^t + \frac{N-i}{i}\left(\frac{N-i-1}{N-1}\right)^t\right),\end{aligned}$$

which concludes the proof. □

Theorem (Out-degree distribution)**.** *At equilibrium, the nodes' expected out-degree $\mathbb{E}\left[d_N^{\text{out},\star}\right]$ in a network of $N \geq 2$ agents equals the $(N-1)$-th harmonic number:*

$$\mathbb{E}\left[d_N^{\text{out},\star}\right] = \sum_{k=1}^{N-1} \frac{1}{k}.$$

Proof. First, we introduce the following notation $\mathbb{E}[d_N^{out,\star}] = d_N$ for the nodes' expected out-degree in a network of N agents. Here we will denote with A_N^k the event of a general node $i \in \{1, \ldots, N\}$ having out-degree k at equilibrium, in a network of N agents. By definition,

$$d_N = \sum_{k=0}^{N} k\mathsf{P}\left[A_N^k\right] = \sum_{k=0}^{N-1} k\mathsf{P}\left[A_N^k\right], \tag{4}$$

where the last step derives from $\mathsf{P}\left[A_N^N\right] = 0$. Trivially we have $d_1 = 0$, and $d_2 = 1$ because in a network with only two agents, node 2 must follow (only) node 1, and node 1 must follow (only) node 2.

In the next part, we prove the following recursion formula:

$$d_N = \frac{1}{N-1}\left(\sum_{k=1}^{N-1}(d_k+1)\right). \tag{5}$$

Using the definition of d_N (4) and the formula derived in the manuscript, i.e.,

$$\mathsf{P}\left[A_N^k\right] = \frac{1}{N-1}\sum_{l=1}^{N-1}\mathsf{P}\left[A_l^{k-1}\right],$$

we obtain

$$\begin{aligned}d_N &= \sum_{k=0}^{N-1} k\mathsf{P}\left[A_N^k\right] = \sum_{k=0}^{N-1} k\left(\frac{1}{N-1}\sum_{l=1}^{N-1}\mathsf{P}\left[A_l^{k-1}\right]\right) = \\ &= \frac{1}{N-1}\left(\sum_{k=0}^{N-1} k \sum_{l=1}^{N-1}\mathsf{P}\left[A_l^{k-1}\right]\right).\end{aligned} \tag{6}$$

Moreover,

$$\begin{aligned}\sum_{k=0}^{N} k \sum_{l=1}^{N}\mathsf{P}\left[A_l^{k-1}\right] &= \sum_{k=1}^{N} k \sum_{l=1}^{N}\mathsf{P}\left[A_l^{k-1}\right] = \\ &= \sum_{k=1}^{N} k\left(\sum_{l=1}^{N-1}\mathsf{P}\left[A_l^{k-1}\right] + \mathsf{P}\left[A_N^{k-1}\right])\right) = \\ &= \sum_{k=0}^{N} k \sum_{l=1}^{N-1}\mathsf{P}\left[A_l^{k-1}\right] + \sum_{k=0}^{N-1}(1+k)\mathsf{P}\left[A_N^k\right].\end{aligned}$$

Note that, for all N,

$$\sum_{k=0}^{N-1}\mathsf{P}\left[A_N^k\right] = 1, \tag{7}$$

then we can use (4) and (7) to obtain:

$$\begin{aligned}\sum_{k=0}^{N} k \sum_{l=1}^{N}\mathsf{P}\left[A_l^{k-1}\right] &= \sum_{k=0}^{N} k \sum_{l=1}^{N-1}\mathsf{P}\left[A_l^{k-1}\right] + d_N + 1 = \\ &= \sum_{k=0}^{N-1} k \sum_{l=1}^{N-1}\mathsf{P}\left[A_l^{k-1}\right] + d_N + 1,\end{aligned} \tag{8}$$

where, for the last step, we also used the fact that $\mathsf{P}\left[A_l^{N-1}\right] = 0$ for all $l \leq N-1$.

Finally, we can use (6) and (8) to obtain

$$d_N = \frac{1}{N-1}\left(\sum_{k=0}^{N-2} k \sum_{l=1}^{N-2}\mathsf{P}\left[A_l^{k-1}\right] + d_{N-1} + 1\right).$$

Note that, for $N = 2$ the above formula correspond to (5). Instead, for $N > 2$, one can iteratively apply (8) until reaching the initial step. Therefore we have proved the recursive formula:

$$d_N = \frac{1}{N-1}\left(\sum_{k=1}^{N-1}(d_k+1)\right).$$

Next, we show that

$$d_{N+1} = d_N + \frac{1}{N}.$$

Using the recursive formula (5) we have just proved,

$$\begin{aligned}d_{N+1} &= \frac{1}{N}\sum_{k=1}^{N}(d_k+1) = \\ &= \frac{1}{N}\left(\sum_{k=1}^{N-1}(d_k+1) + (d_N+1)\right) = \\ &= \frac{1}{N}\left(\sum_{k=1}^{N-1}(d_k+1) + \frac{1}{N-1}\sum_{k=1}^{N-1}(d_k+1) + 1\right) = \\ &= \frac{1}{N}\left(\frac{N}{N-1}\sum_{k=1}^{N-1}(d_k+1) + 1\right) = \\ &= \frac{1}{N-1}\sum_{k=1}^{N-1}(d_k+1) + \frac{1}{N} = \\ &= d_N + \frac{1}{N}.\end{aligned}$$

Finally note that for $N = 2$, $d_N = 1 = \sum_{k=1}^{N-1} \frac{1}{i}$. Assume that $d_{N'} = \sum_{k=1}^{N'-1} \frac{1}{i}$ for all $N' < N$, then by induction we prove that

$$d_N = d_{N-1} + \frac{1}{N-1} = \sum_{k=1}^{N-2} \frac{1}{i} + \frac{1}{N-1} = \sum_{k=1}^{N-1} \frac{1}{i},$$

which concludes the proof. □

Special case: Bipartite-like networks

In many real-world online platforms, e.g., YouTube and Twitch, the users can be roughly partitioned into two classes: the influencers, who actively contribute content, and the followers, who never generate any content but only follow the influencers. For these online platforms, the underlying networks can be approximated by a special bipartite-like structure: all the directed links are either among the influencers or from the followers to the influencers. By assigning zero quality to the followers, our quality-based model can be directly applied to explain the formation of such bipartite-like network structure.

Consider the quality-based model defined in the main text with $m+n$ individuals, consisting of m influencers and n followers. The individual quality attribute of each influencer $r \in \{1,\dots,m\}$ is characterized by a parameter $q_r > 0$. The quality q_i for any follower $i \in \{m+1,\dots,m+n\}$ is set to be 0. Suppose that, at time $t = 0$, the payoff of each individual $j \in \{1,\dots,m+n\}$, i.e., the maximal quality $V_j(0)$ received by j, is 0.

The discrete-time stochastic process described above almost surely terminates at finite time, since every individual will eventually meet and follow the influencer with the highest quality, after which no link will be built. Moreover, according to equation (3) in the main text, no link will ever be built from an influencer to a follower, or from a follower to another follower. Therefore, the presence of the followers does not affect the links built by the influencers.

In-degree distribution: Apparently, since the followers all have zero quality, no link will ever be built from any node to any follower. Therefore, the in-degree of any follower is zero. Results on the in-degree distribution for the influencers are given as follows.

Theorem (In-degree distribution for influencers)**.** *Consider a group of m influencers, indexed by $r \in \{1,\dots,m\}$, and n followers. Without loss of generality, suppose that the influencers' qualities are ranked as $q_1 > q_2 > \cdots > q_m$. For any given follower, the probability that she follows any influencer r is given by $1/r$. Moreover, the expected in-degree for any influencer $r \in \{1,\dots,m\}$ is equal to $(n+m)/r$ for any $r > 1$ and equal to $n+m-1$ for $r = 1$.*

Proof. For any given follower, suppose the $T_{\leq r}$-th link she builds is their first link to an influencer in the set $\{1,2,\dots,r\}$. Apparently, $T_{\leq r}$ is a random variable. By definition of the network formation process, we have $T_{\leq r} \leq m-r+1$. Here the equality corresponds to the event that this follower build their first $m-r$ links to influencers $m, m-1, \dots, r+1$ consecutively and then build the $(m-r+1)-$th link to some influencer in the set $\{1, 2, \dots, r\}$.

For this given follower, define the random variable V_t as the index of the influencer she builds their t-th link to. According to the network formation process, since the follower will no longer follow influencer r if she builds their $T_{\leq r}$-th link to some influencer $s \in \{1,\dots,r-1\}$, we have

$$\begin{aligned}
&\mathbb{P}[\text{Influencer } r \text{ is followed by this follower}] = \\
&= \mathbb{P}[V_{T_{\leq r}} = r] \\
&= \sum_{t=1}^{m-r+1} \mathbb{P}[V_t = r \,|\, T_{\leq r} = t]\mathbb{P}[T_{\leq r} = t] \\
&= \sum_{t=1}^{m-r+1} \frac{1}{r}\mathbb{P}[T_{\leq r} = t] \\
&= \frac{1}{r}.
\end{aligned}$$

Now suppose there are n followers. Since the actions of the followers are independent, the probability that any influencer $r \in \{1,2,\dots,m\}$ is followed by k followers is given by

$$\binom{n}{k}\left(\frac{1}{r}\right)^k\left(1-\frac{1}{r}\right)^{n-k}$$

and

$$\begin{aligned}
&\mathbb{E}[\text{Number of followers following } r \text{ eventually}] \\
&= \sum_{k=0}^{n} k\binom{n}{k}\left(\frac{1}{r}\right)^k\left(1-\frac{1}{r}\right)^{n-k} = \frac{n}{r}.
\end{aligned}$$

Besides the links from followers, each influencer may also receive some links from other influencers. The number of in-links from other influencers is independent of the number of followers but depends on the number of influencers in total. According to equation (8) in the main text,

$$\begin{aligned}
&\mathbb{E}[\text{Number of influencers following } r \text{ eventually}] \\
&= \begin{cases} m-1, & \text{if } r = 1, \\ m/r, & \text{otherwise.} \end{cases}
\end{aligned}$$

Therefore, the expected in-degree of any influencer r is given by

$$\begin{aligned}
&\mathbb{E}[\text{The in-degree of influencer } r] \\
&= \mathbb{E}[\text{Number of followers following } r \text{ eventually}] \\
&\quad + \mathbb{E}[\text{Number of influencers following } r \text{ eventually}] \\
&= \begin{cases} n+m-1, & \text{if } r = 1, \\ (n+m)/r, & \text{otherwise.} \end{cases}
\end{aligned}$$

This concludes the proof. □

Note that, when the number of followers n is much larger than the number of influencers m, then the above results approaches the one obtained in the manuscript for the general case, with

$n \sim N = n + m$. In this case, the ties created by the influencers only marginally affect the results, and thus the influencers in-degree distribution could also be studied considering the network as a perfectly directed bipartite network, in which the ties are only from the followers to the influencers.

Out-degree distribution: Since the followers have zero quality, they have no effect on the behavior of the influencers. That is, the influencers build links to other influencers as if those followers did not exist. Therefore, the out-degree distribution for the influencers is still given by equation (9) in the main text, i.e., the expected out-degree equals $H(m-1)$, where $H(\cdot)$ is the harmonic number function. As for the followers, their out-degree distribution is given as follows.

Theorem (Out-degree distribution for followers). *Denote by p_m^k the probability that a follower eventually follows k influencers when there are m influencers in total. We have*

(a) $p_m^k = 0$ *as long as* $m < k$*;*

(b) $p_m^1 = 1/m$ *and* $p_m^m = 1/(m!)$ *for any* $m \in \mathbb{N}_+$*;*

(c) For any $m, k \in \mathbb{N}_+$ *with* $2 \le k \le m$, p_m^k *satisfies the following iteration equation:*

$$p_m^k = \frac{1}{m} \sum_{r=1}^{m} p_{r-1}^{k-1}.$$

(d) Moreover, with m influencers in total, for each follower, the expected number of followees is given by

$$\mathbb{E}[\textit{number of followees} \,|\, \textit{there are m influencers}] = 1 + \frac{1}{2} + \cdots + \frac{1}{m}.$$

Proof. Without loss of generality, assumed that there are m influencers in total and they are indexed such that $q_m < q_{m-1} < \cdots < q_1$.

Statement (a) holds by definition. Regarding statement (b), a follower only has one followee at the final steady state if and only if the first influencer this follower meets happens to be the influencer with the highest quality, of which the probability is $1/m$, i.e., $p_m^1 = 1/m$. For any follower, p_m^m is the probability of the event that she first meets influencer m, and then meets influencer $m-1$, and then meets $m-2$, and so on. The corresponding probability is $(1/m) \cdot (1/(m-1)) \cdot \cdots \cdot (1/2) \cdot 1 = 1/m!$. This concludes the proof of statement (b).

For any given follower i and any $2 \le k \le m$, by probability theory, the following equalities hold:

$$\begin{aligned}\mathbb{P}[&i \text{ follows } k \text{ influencers} \,|\, \text{there are } m \text{ influencers}] \\ &= \sum_{r=1}^{m} \mathbb{P}[i \text{ follows } k \text{ influencers} \,|\, i \text{ first meets } r, \text{there are } m \text{ influencers}] \\ &\quad \cdot \mathbb{P}[i \text{ first meets } r \,|\, \text{there are } m \text{ influencers}]\end{aligned}$$

The event

$$i \text{ follows } k \text{ influencers} \,|\, i \text{ first meets } r, \text{there are } m \text{ influencers}$$

is equivalent to the event

$$i \text{ follows } k-1 \text{ influencers} \,|\, \text{there are } r-1 \text{ influencers}$$

and the latter occurs with probability p_{r-1}^{k-1}. In addition,

$$\mathbb{P}[i \text{ first meets } r \,|\, \text{there are } m \text{ influencers}] = 1/m.$$

Therefore,

$$\mathbb{P}[i \text{ follows } k \text{ influencers} \,|\, \text{there are } m \text{ influencers}] = \sum_{r=1}^{m} \frac{1}{m} p_{r-1}^{k-1}.$$

This concludes the proof of statement (c).

For any given follower $i \in \{n+1, \ldots, n+m\}$, let the random variable K be the number of influencers i follows. By probability theory,

$$\begin{aligned}&\mathbb{E}[K \,|\, \text{there are } m \text{ influencers}] \\ &= \sum_{r=1}^{m} \mathbb{P}[i \text{ first meets } r \,|\, \text{there are } m \text{ influencers}] \\ &\qquad \cdot \mathbb{E}[K \,|\, i \text{ first meets } r, \text{there are } m \text{ influencers}] \\ &= \sum_{r=1}^{m} \frac{1}{m} \Big(1 + \mathbb{E}[K \,|\, \text{there are } r-1 \text{ influencers}]\Big)\end{aligned}$$

Let $k_s = \mathbb{E}[K \,|\, \text{there are } s \text{ influencers}]$ for any $s \in \mathbb{N}$. Apparently, $k_0 = 0$. Since

$$k_m = \frac{1}{m} \sum_{r=1}^{m} (1 + k_{r-1}) = 1 + \sum_{r=1}^{m} k_{r-1},$$

we have $k_s = k_{s-1} + 1/s$, which implies that

$$k_m = 1 + \frac{1}{2} + \cdots + \frac{1}{m}.$$

This concludes the proof of statement (d). □

The expected out-degree for any follower is $H(m)$, which is not very different from $H(m-1)$, i.e., the expected out-degree for any influencer. As such, the above result corresponds to the one obtained for general networks, with the difference that the expected out-degree depends only on the number of influencers, and not on the number of followers (which cannot be followed, by construction).

Supplementary Note 2. *Numerical analysis*

Preferential Attachment meeting process

In the theoretical results presented in the manuscript, the meeting process was based on a uniform probability density function such that every user had the same probability of finding any

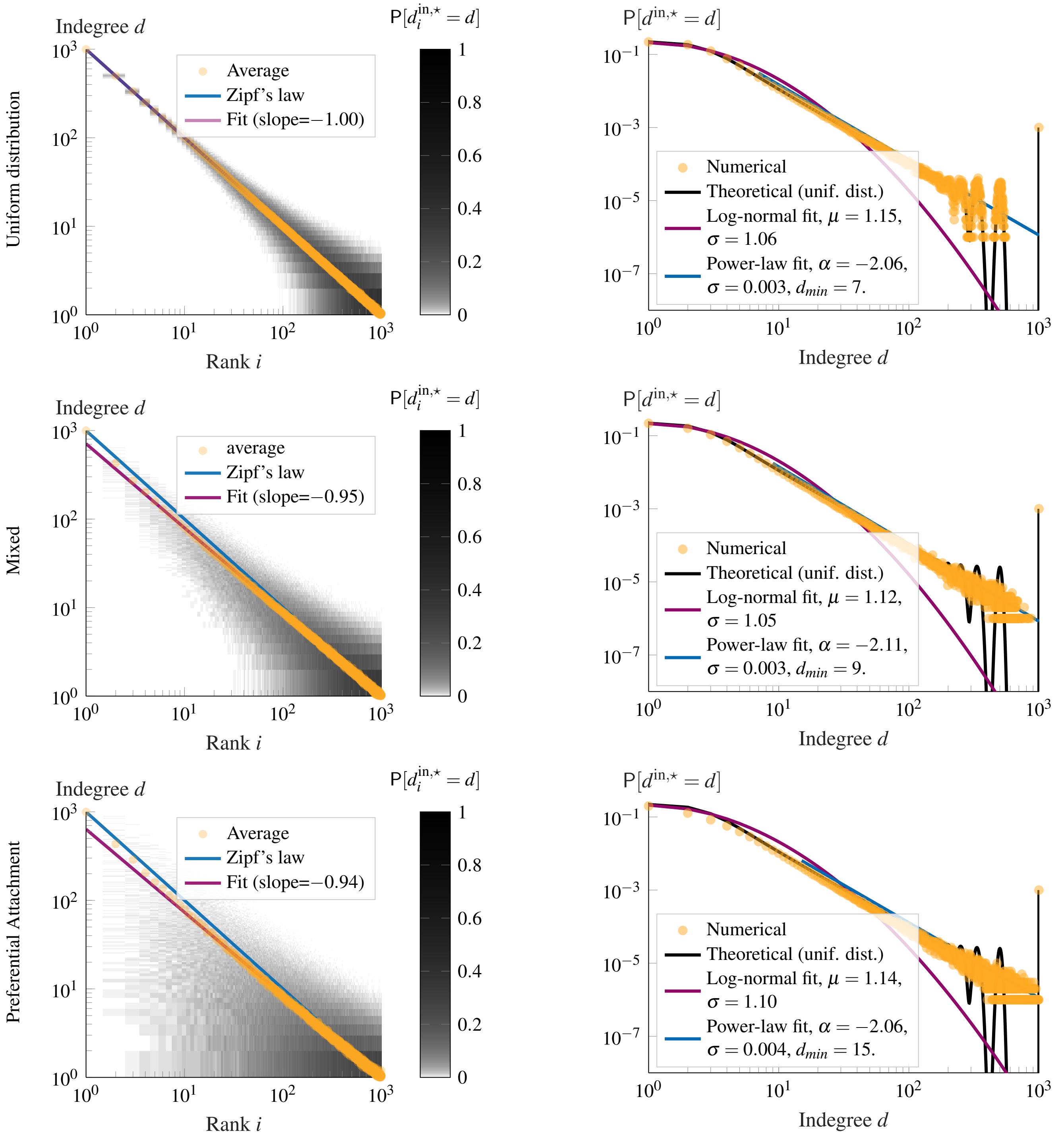

Supplementary Figure 1. Numerical results of 1000 simulations with 1000 nodes for three different meeting process scenarios (top: uniform distribution, bottom: pure preferential attachment, middle: mixed). On the left, the color maps show the in-degree probability density function, as a function of the quality rank. In orange, the average in-degree. In blue, the Zipf's law which equals the expected in-degree in the uniform distribution scenario. In purple, the linear fit (in log-log) of the average values. On the right, the numerical probability density functions (orange) and their power-laws (blue) and log-normal (purple) fits. In black, the theoretical results for the scenario with uniform probability distribution is plot as reference.

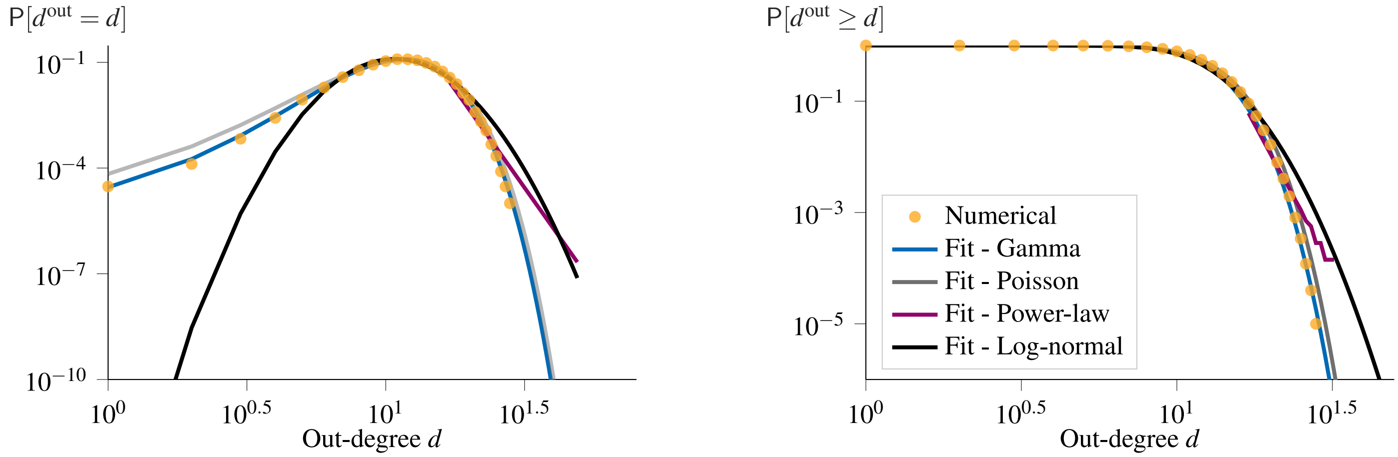

Supplementary Figure 2. Results from a network of 10^5 nodes: on the left, the out-degree probability density function, on the right the complementary cumulative distribution function. The Gamma distribution is best fitted with parameters: $\alpha = 47.71$, $loc = -10.24$, $\beta = 0.47$. The Poisson distribution is fitted with parameter λ equal to the Harmonic number of $N-1$, the power-law with parameters $\alpha = -11.11 \pm 0.11$, $d_{min} = 17$, and the log-normal with parameters $\mu = 2.45$, $\sigma = 0.28$.

other user. In this section, we study a meeting process in which users' probability to be met is proportional to their current in-degree, as in the preferential attachment model[1]. By doing so, we try to mimic the effect of the recommendation systems which may increase the probability of being exposed to the content of high in-degree nodes. On the other hand, though, we leave the original individual linking decision-making untouched, i.e., agent i follows a new agent j only if the quality of j increases the utility function of agent i (which is measured by the maximum quality of the content i is exposed to through his/her followees).

To quantify the effect of this preferential attachment based meeting process, we compare three different scenarios. In the first one, we use a uniform probability distribution, in the second one, we equally mix uniform probability distribution and preferential attachment, and in the third one, we only use preferential attachment (based on the in-degree of the nodes). Since a theoretical analysis of the second and third scenarios is increasingly complex, we run 1000 Monte Carlo simulations (per scenario) and generate the corresponding equilibrium networks.

On the left column of Supplementary fig. 1, we present the results on the rank vs in-degree plot. Noticeably, introducing the (full or mixed) preferential attachment process increases the variance in the in-degree probability distribution of each agent. For instance, especially in the third scenario, it is not unlikely that some of the high-quality nodes (e.g., in the top 50) receive less than 10 followers (which, instead, has almost zero probability in the uniform distribution scenario). In other words, the preferential attachment may penalize some agents, which receive fewer followers than what they would expect with the uniform distribution process (whose theoretical average corresponds to the Zipf's law). On the other hand, it is also possible that some low-quality agents get an initial advantage (purely by chance), which gets reinforced by the preferential attachment mechanism. Yet, this effect quickly fades away, because potential followers still undergo the quality threshold rule. Interestingly, though, the average number of followers as a function of the ranking is close to the Zipf's law, even in the case of full or mixed preferential attachment. The coefficient of the fitted line goes from -1 in the uniform distribution case, to -0.94 in the pure preferential attachment. Moreover, it is remarkable that, on average, the meritocratic principle remains satisfied even after introducing the preferential attachment process. In fact, while the increasing variance of the probability distribution may more frequently result in lower quality nodes having higher in-degree than higher quality ones, the correlation between quality and followers persists: the higher the quality, the higher the average number of followers.

In summary, the introduction of a preferential attachment based meeting process can partially alter the dynamics. However, per the effect of the meritocratic principle in the update rule, the final results in terms of in-degree vs quality ranking remain confirmed (on average). Deviations from this average, though, can be more likely than with a uniform distribution meeting process.

Out-degree distribution

Even though the theoretical out-degree distribution cannot be directly associated to any known distribution, it is very close to a gamma distribution (or to a Poisson distribution), as shown in Supplementary fig. 2. According to the results of the Kolmogorov-Smirnov test, the distance between the empirical distribution and the gamma and Poisson distributions are, respectively, 0.06 and 0.13. Thanks to its non-monotonic nature and its very fast decrease, there are some similarities with the log-normal distributions, while it differs more significantly from a power-law distribution (the distance between the empirical distribution with the log-normal is 0.49, and with the power-law is 0.97).

The behavior of a gamma (or a Poisson) distribution is compatible with the cut-off (artificial or not) which is present in empirical out-degree distribution, e.g., on Instagram[2,3] or YouTube[4]. Moreover, the non-monotonic feature is also compatible with the fact

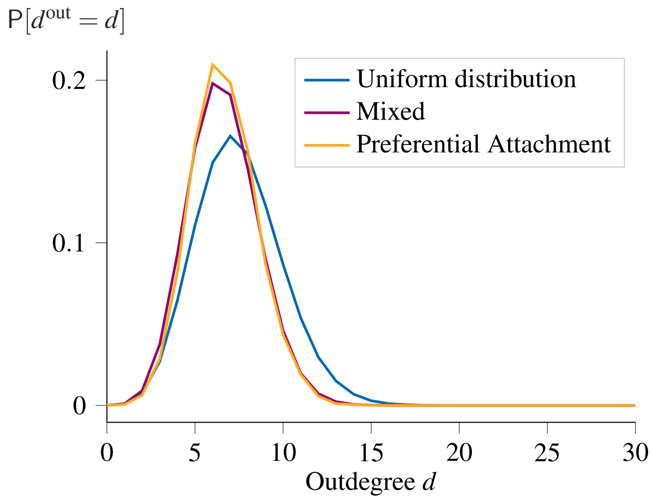

Supplementary Figure 3. Numerical results of the empirical out-degree distribution of 1000 simulations of equilibrium networks of 1000 nodes, for the three different scenarios: uniform distribution, pure preferential attachment process, and mixed approach.

that the majority of (active) users on these social networks have at least (and at most) $10^1 - 10^2$ out-connections, as shown in the empirical Instagram data-sets of[2,3]. In other words, the out-degree complementary cumulative distribution function is substantially flat until reaching $d = 10^1 - 10^2$, as shown in Supplementary fig. 2.

To complement our analysis on the out-degree distribution, we also study the effect of the preferential attachment meeting process considering 1000 network realizations of 1000 nodes. From the comparison depicted in Supplementary fig. 3, we evince that the preferential attachment tends to shift the probability density function to the left, but the shape of the distribution remains unvaried.

Clustering coefficient

For the same three different scenarios of the meeting process, we also studied the average clustering coefficient as a function of the network size. The results are shown in Supplementary fig. 4, and for each value of N we run 1000 simulations. While a monotonic decrease of the average clustering coefficient can be noticed for all three scenarios, only minor differences are noticeable across the scenarios. Ultimately, in the presence of (full or partial) preferential attachment, slightly larger values of average clustering coefficients are observed.

Overlap Index

As anticipated, we offer an intuition for the behavior observed in the audience overlap index in fig. 10 in the manuscript. In the sketch in Supplementary fig. 5 we show three different possible realizations of the followers' sets of the nodes ranking $1-3$, for a network of general size N. In all these cases, the size of the followers' sets equals the expected values, as in eq. (8) (of the manuscript), i.e., $|\mathscr{F}_2^{\text{in}}| = |\mathscr{F}_1^{\text{in}}|/2$ and $|\mathscr{F}_3^{\text{in}}| = |\mathscr{F}_1^{\text{in}}|/3$. As one can easily verify, for all the cases, $O(i,1) = 1$ and $O(1,j) = 1/j$.

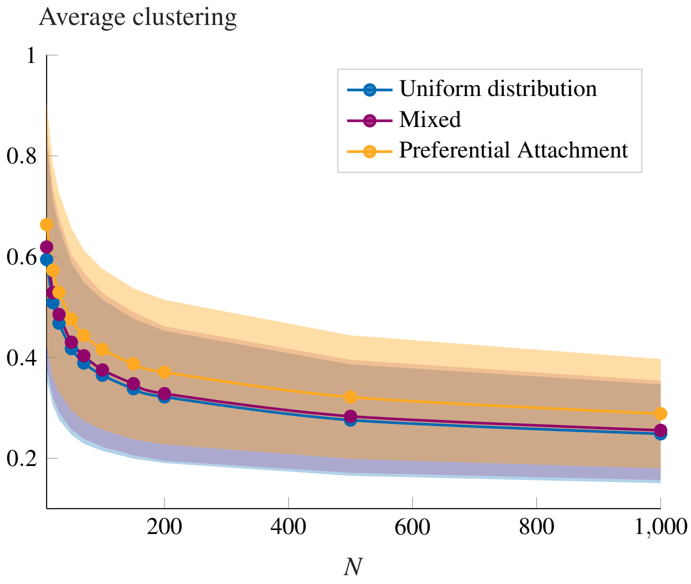

Supplementary Figure 4. Numerical analysis of the average clustering coefficient for different network sizes.For each value of N, we run 1000 simulations, in three different settings for the meeting process probability distribution: in blue, we use a uniform distribution, in orange, we use a preferential attachment mechanism, and in purple we use a mixed distribution (50% chance of uniform distribution and 50% chance of preferential attachment, for each meeting). The data-points indicate the average value. The shaded area indicates one standard deviation.

Moreover, from left to right we have $O(2,3)$ equals to $1/3, 2/3, 0$ respectively, and $O(3,2)$ equals to $1/2, 1, 0$, respectively. Since the three scenarios happen with the same probability (because each user's dynamic is an independent process), we obtain the average values of $O(2,3) = 1/3$ and $O(3,2) = 1/2$, i.e., Zipf's law. Of course, the possible scenarios are more than the three sketched, and the sets can possibly have sizes which differ from the expected values. However, when averaging upon a sufficiently large number of realizations the audience overlap index satisfies the Zipf's regularity in every row, as in fig. 10 in the manuscript.

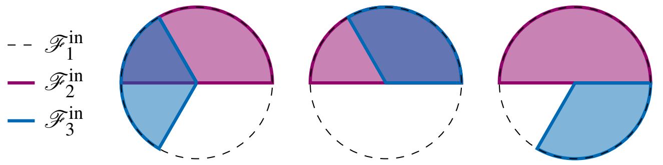

Supplementary Figure 5. Sketch of the possible overlap between followers' sets.

Equal quality test

In this example, we study the impact of having two or more users of the same quality. In particular, we consider (and compare) two scenarios: in the first scenario (i), users follow the same dynamics as the one expressed in eq. (3) in the manuscript. In this case, if there are two or more users of the same quality, following one of them prevents from following the other(s). In the second scenario

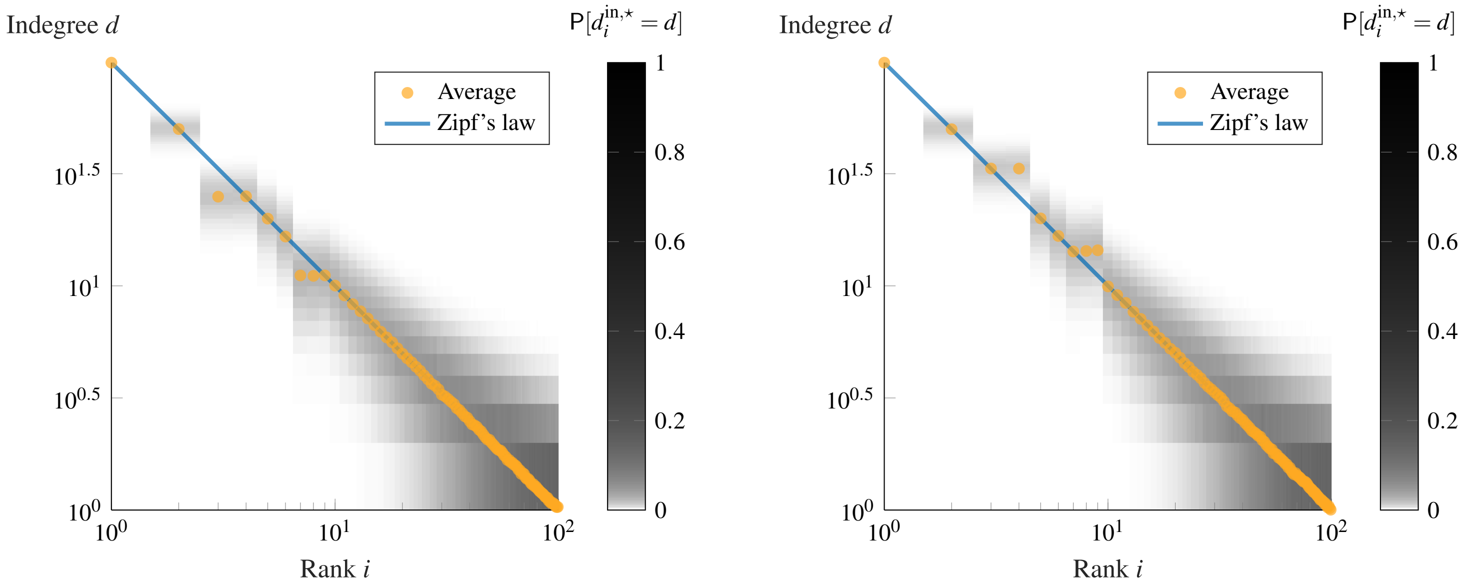

Supplementary Figure 6. Results for the equal quality test. On the left, we tested the (i) scenario, on the right the (ii) scenario. For both scenarios, we plot the empirical in-degree probability density functions (as a function of the node rank) resulting from 10′000 simulations (upon reaching convergence) for networks of $N = 100$ nodes. For this test, $q_3 = q_4$ and $q_6 = q_7 = q_8$. In blue, we plot the Zipf's law N/i, which corresponds to the expected in-degree when no equality nodes are present.

(ii), we consider a modified dynamics, i.e.,

$$a_{ij}(t+1) = \begin{cases} 1, & \text{if } q_j \geq V_i(t), \\ a_{ij}(t), & \text{otherwise,} \end{cases}$$

in which we introduce a non-strict inequality condition. Such a modification does not yield any change when the users have all different qualities. However, now, if there are 2 or more users of the same quality, following one of them does not prevent following the other(s). We test the two scenarios on 10′000 simulations of a network of $N = 100$ nodes in which the quality of the node 3 and 4 is forced to be the same. Similarly, the quality of nodes 6, 7, and 8 is the same. The results of the two scenarios are presented in Supplementary fig. 6.

First of all, we notice that the other nodes are not affected, and their in-degree probability density functions (as well as their average in-degree) are the same as in the situation without equal-quality nodes, i.e., correspond to the Zipf's law. On the other hand, the in-degree probability density functions are similar across agents of the same quality. The same applies to their average in-degree, thus the meritocratic principle is preserved. The only difference between the two scenarios is as follows: in (i), the average in-degree equals the Zipf's value computed at the lowest ranking node, i.e., $N/4$ for nodes 3 and 4, and $N/8$ for nodes 6, 7, and 8. Conversely, in (ii), the average in-degree is $N/3$ for nodes 3 and 4, and $N/6$ for nodes 6, 7, and 8. In summary, the nodes of equal quality remain on the left (respectively, right) of the Zipf's law in the first (respectively, second) scenario, while all other nodes satisfy the Zipf's law.

With the above example, we illustrated that the difference is marginally small and limited to the prediction of those users.

Supplementary Note 3. *Twitch data collection*

Twitch is an online social media platform focusing on video streaming, including broadcasts of gameplay, e-sports competitions, and real-life content. Since its launch in 2011, Twitch has gradually become one of the most popular social media platforms, and it is particularly diffused among the young generations: more than half of the audience is aged between 18 and 34, and 14% is between 13 and 18[5]. Similarly to YouTube, the UGC on Twitch is in the form of videos, and more precisely of live-streamed videos. Users can create dedicated channels to stream their performances that others can watch and follow. Given that only a minority of the users provide UGC, we distinguish between *streamers*, or *broadcasters*, i.e., users that produce UGC, and *viewers*, i.e., followers that consume the UGC of the streamers.

According to the topic, the content is classified into appropriate categories. Some of them are broad categories, e.g., art and music, while others are more focused, e.g., Fortnite or Minecraft (which are popular e-games). Categories help users to browse the content of their interest and to discover new streamers to follow. At the same time, categories are helpful in the identification of network communities, i.e., groups of users (streamers and their followers) with the same interest. Given the assumptions of our model, this characteristic is particularly convenient, as it simplifies the restriction of the analysis to a subset of similar users. Moreover, similar to Twitter, Twitch has the advantage that it provides its own API[6], which allows to efficiently collect data about users and their underlying interconnections. Compared to Twitter, though, Twitch API has much fewer restrictions in terms of rate-limits for web-crawling, thus it is more suitable for collecting large

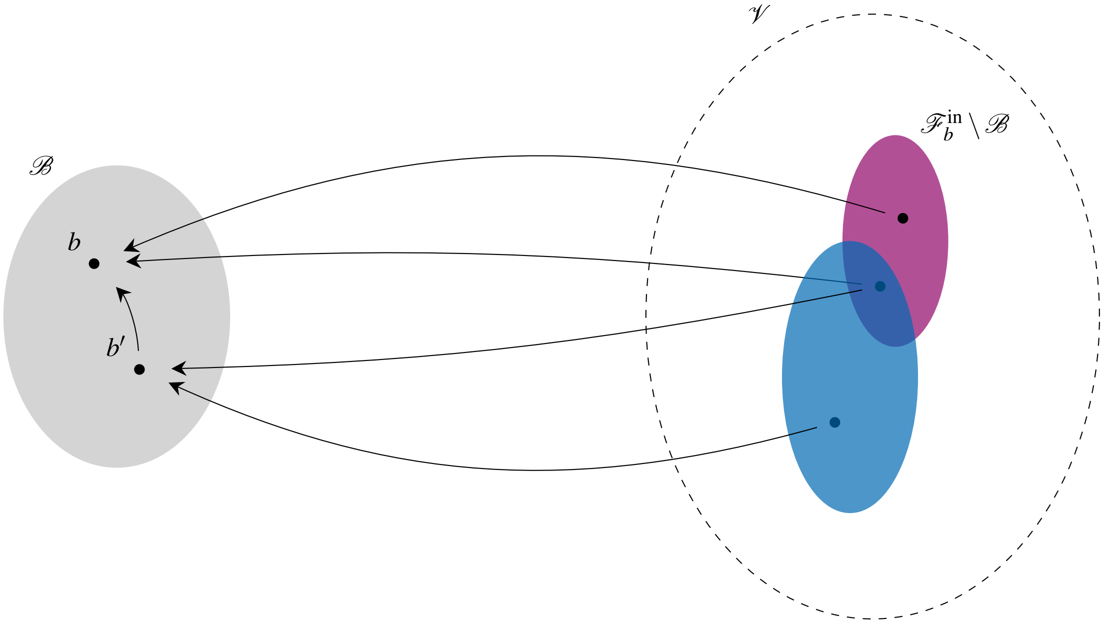

Supplementary Figure 7. Sketch of the bipartite-like network composed of the set of broadcasters $\mathscr{B}$ (on the left) and of viewers $\mathscr{V}$ (on the right). For each broadcaster b, we crawl the entire set of followers $\mathscr{F}_b^{\rm in}$. Clearly, followers' sets can overlap, and broadcasters can be followed by other broadcasters.

data-sets.

Category identification

In order to validate our model results, we need to collect data-sets that fulfill our assumptions. In particular, we need to find a set of users with a common interest, and to reconstruct the network of relationships among them. To do so, we first need to identify categories that define a stable community of interested users, over time.

On Twitch, more than a thousand different games are streamed simultaneously. Many new games are launched every day, and users are likely to be attracted by them. Yet, most online games tend to have a short life-time, lasting only for a few months. Unlike them, more traditional games such as poker or chess attracted people from all over the world for centuries. Similarly, categories such as art are intuitively less subject to a change of user's interest.

To support our intuition we looked at the historical trend of viewers in the category poker, shown in Supplementary fig. 8. Until 2019, the monthly amount of viewers shows a stationary trend (except for periodic annual oscillations), indicating that the poker category is not sensitive to user's interest lability. From the comparison with several other categories, the growth during 2020 should not suggest a recent raising interest in the game of poker. Rather, it must be seen as a side effect of the quarantines due to the Covid-19 pandemic, which triggered a generalized increase in the usage of the Twitch platform[7]. Qualitatively similar results are obtained for the chess and art categories, hence they all represent suitable choices for our data-sets collection.

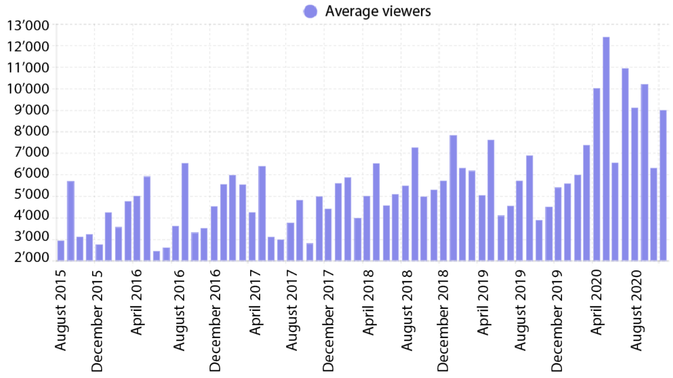

Supplementary Figure 8. Average viewers in the poker category of Twitch. Source:[8].

Crawling procedure

After selecting the categories, we then set up the crawling of the corresponding Twitch data-sets. For each of these categories then:

- we crawl the set of all the broadcasters, or streamers, denoted with $\mathscr{B}$, that "consistently" stream into that category;
- for each user $b \in \mathscr{B}$, we crawl the set of followers $\mathscr{F}_b^{\rm in}$ of b. Among them we find other broadcasters $b' \in \mathscr{B}$, but also other users (viewers) which do not produce any UGC. We denote the union of the set of viewers as $\mathscr{V} := \cup_b \mathscr{F}_b^{\rm in} \setminus \mathscr{B}$.
- finally, we construct the bipartite-like network where the set of nodes $\mathscr{N}$ is given by $\mathscr{N} = \mathscr{B} \cup \mathscr{V}$, and the ties are either within $\mathscr{B}$ or directed from $\mathscr{V}$ to $\mathscr{B}$, as sketched in Supplementary fig. 7.

Note that, since among the followers $\mathscr{F}_b^{\text{in}}$ of each broadcaster b there could also be $b' \in \mathscr{B}$, the network is not a perfectly bipartite network (see the discussion in Supplementary Note 1). However, given that $|\mathscr{B}| \ll |\mathscr{V}|$, i.e., the number of broadcasters is negligible with respect to the number of viewers, the majority of the ties are directed from $\mathscr{V}$ to $\mathscr{B}$, and the network is almost a bipartite network.

Twitch is a multilingual platform, and broadcasters using different languages target different audiences, therefore they will unlikely form a single community. Rather, they might form a network that can be easily partitioned according to the language. Hence, we decide to restrict our crawling to the data concerning the users streaming in English, which is the most represented language, as shown for the chess category in Supplementary fig. 9. We emphasize that, on Twitch, it is possible to crawl data only on the users that are currently live-streaming. Thus, we repeat our crawling every hour for a period of one week until reaching a stable and consistent ranking in the list of the top 30 most followed broadcasters. In the end, we find 492, 547, and 5086 unique users streaming, respectively, in the chess, poker, and art category.

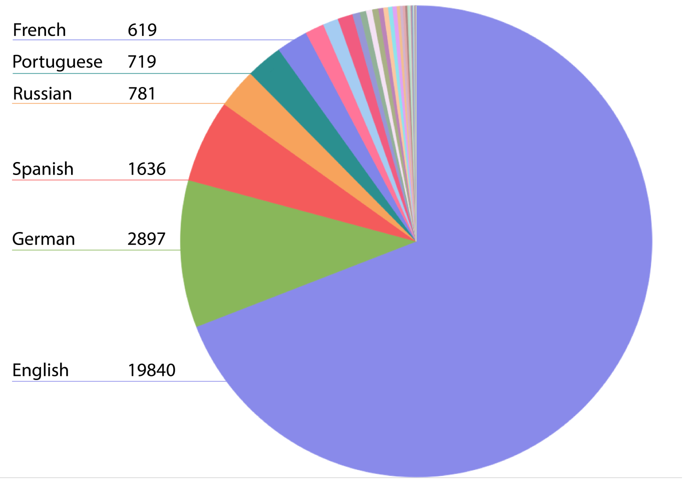

Supplementary Figure 9. Language of broadcasters which played chess between September 2019 and September 2020. Source:[9].

Once we collect the broadcasters' sets $\mathscr{B}$ (one for each category), we then use the Twitch API to crawl the broadcasters' followers, and we finally construct the bipartite-like network. We do not crawl the information on the ties between the followers, as they do not provide any UGC.

Interest index

Crawling all the (live) broadcasters in one category, e.g., chess, guarantees that they have an interest in the game of chess. However, for some of them, this may only be a secondary interest. According to our modeling assumptions, the network formation process is based on the shared user's interest in a specific topic. One consequence of this can be found in the high overlap among the followers' sets. Reconstructing the underlying network suffers from this multi-interest effect, where the follower relationship might be due to a different interest. In that case, the followers of a certain broadcaster may present a very low overlap with the followers of the other broadcasters. Evidence of this is shown in Supplementary fig. 11a, depicting the audience overlap index (as defined in the manuscript) among the top 20 broadcasters in the chess data-set. Clearly, some users (ranked 12, 16, and 17) have very low followers' sets intersections with the other top broadcasters.

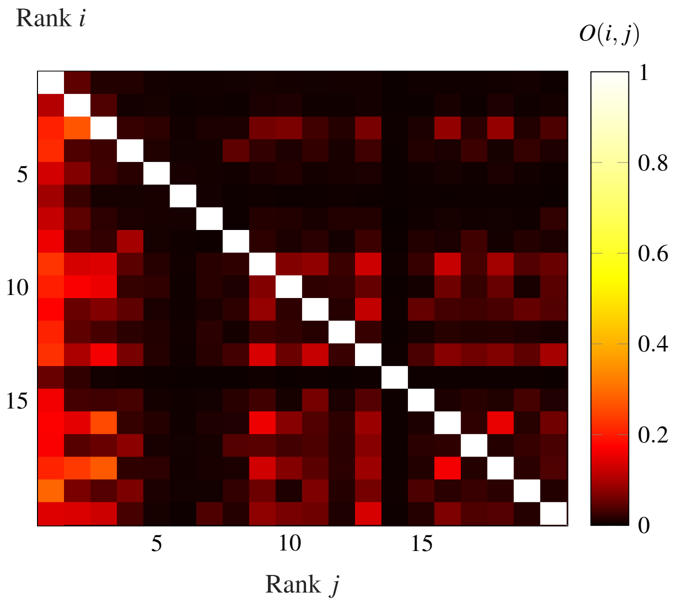

Supplementary Figure 10. Followers' overlap results among the top 20 nodes in the art data-set, after applying the "interest" index criterion. The result is quantitatively very different from the one presented in Supplementary fig. 11b, denoting that the broadcasters in the art category do not share followers from the same community.

In order to reduce the impact of the multi-interest effect, we use a filtering criterion based on the amount of time users spend on streaming on that given category. Practically, we only consider the broadcasters that "consistently" stream into the chosen category, i.e., for at least 80% of their streaming time. The intuition is that this criterion should primarily affect those broadcasters that spend a non-negligible part of their streaming time into different categories, thus accumulating a diversified audience. Note that a consistent part of the original broadcasters sets is retained: among the original 492, 547, and 5086 broadcasters in the chess, poker, and art categories, respectively 305, 358, and 2332 of them satisfy the criterion. Yet, setting such a minimum threshold leads to a generalized improvement in the followers' overlap matrix for the chess data-set, as illustrated in Supplementary fig. 11b. Similar results are obtained for the poker data-set. On the other hand, the result concerning the art data-set, pictured in Supplementary fig. 10, indicates that the overlap among the followers' sets remains generally very low. One possible explanation for this effect can be found in the extreme generality of this category. While both chess and poker precisely target a community of users with an interest in a specific game, the art category comprises users that may be attracted by different styles. Thus, each broadcaster's

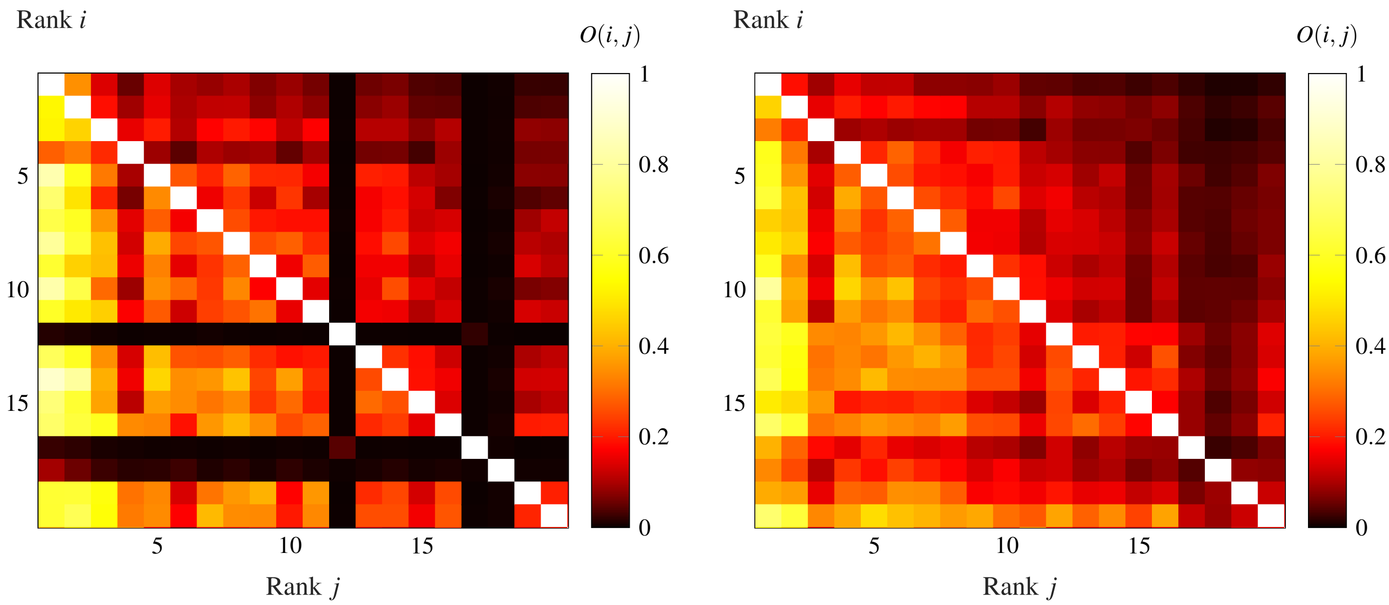

(a) **(b)**

Supplementary Figure 11. Followers' overlap results among the top 20 nodes in the chess data-set. In **(a)**, the broadcasters are the pure raw data. In **(b)**, we first applied the filtering criterion on the "interest" index that results in the exclusion of some broadcasters (ranked 12, 16, and 17 on the left) with very low followers' overlap.

followers set has very limited overlap with the others.

The different performance in terms of audience overlap index of the three data-sets highlights the importance of having a baseline community of interested users. Apparently, the network formation process related to the art category data-set is significantly different from that of the quality-based model, therefore we focus our data analysis on the other two data-sets: poker and chess.

Supplementary Note 4. *Twitch data analysis*

In this section, we report some additional empirical analysis of the Twitch data-sets. In particular, we first study the in-degree distribution of the broadcasters, then the out-degree distribution of the followers, and finally the audience overlap between the top-quality broadcasters.

Broadcasters in-degree distribution

In the manuscript, we have already shown that the empirical in-degree vs. rank data satisfy the Zips' law, as predicted by our model. In Supplementary fig. 12 we show the histograms of the Complementary Cumulative (top) and probability density (bottom) empirical distributions for the chess data-set (similar results are obtained for the poker data-set), and we compare them with different fitting functions.

We find that the log-normal fit (in orange) is a good approximation when focusing on the low in-degree nodes region. On the other hand, the log-normal distribution deviates substantially from the empirical data in the high in-degree nodes region.

We also used two different power-laws to fit the empirical data. In purple, a power-law of parameter $\alpha = 1.72$ (and $d_{min} = 1425$) is the best fit (in terms of log-likelihood). While it provides a good description of the middle-ranking nodes, its slope is not a good fit for the high in-degree nodes. A slightly better power-law fit can be obtained by forcing $d_{min} = 18000$ (blue line). In this case, the coefficient is very close to $\alpha = 2$, and the fit captures the scaling of the high in-degree nodes.

Finally, in black, we plot a pure Zipf's law, in which each node has in-degree proportional to the inverse of its ranking. Note that the pure Zipf's law used is a computationally tractable approximation of our theoretical results: in our analysis, each node follows the Zipf's law *only* in expectation. Yet, such a distribution is a good approximation of our theoretical probability distribution function in the region of high in-degree nodes, as shown in Supplementary fig. 13, while it deviates marginally in the region of low in-degree nodes. In this case, the Zipf's law correctly captures the trend of the high in-degree nodes, which tend to follow the sequence $N, N/2, N/3, \ldots$. On the other hand, as mentioned in the Methods section, the empirical in-degree distribution is likely to be affected by a sampling bias due to the fact that we can only fetch the broadcasters that were actively broadcasting during our data collection period. Luckily, such a sampling bias only affects the region of low in-degree nodes, but not the most popular nodes.

In summary, our theoretical distribution is the only one that predicts and captures the Zipf's sequence which is typical of the high in-degree nodes. On the other hand, the log-normal seems to provide a better fit in the region of low in-degree nodes.

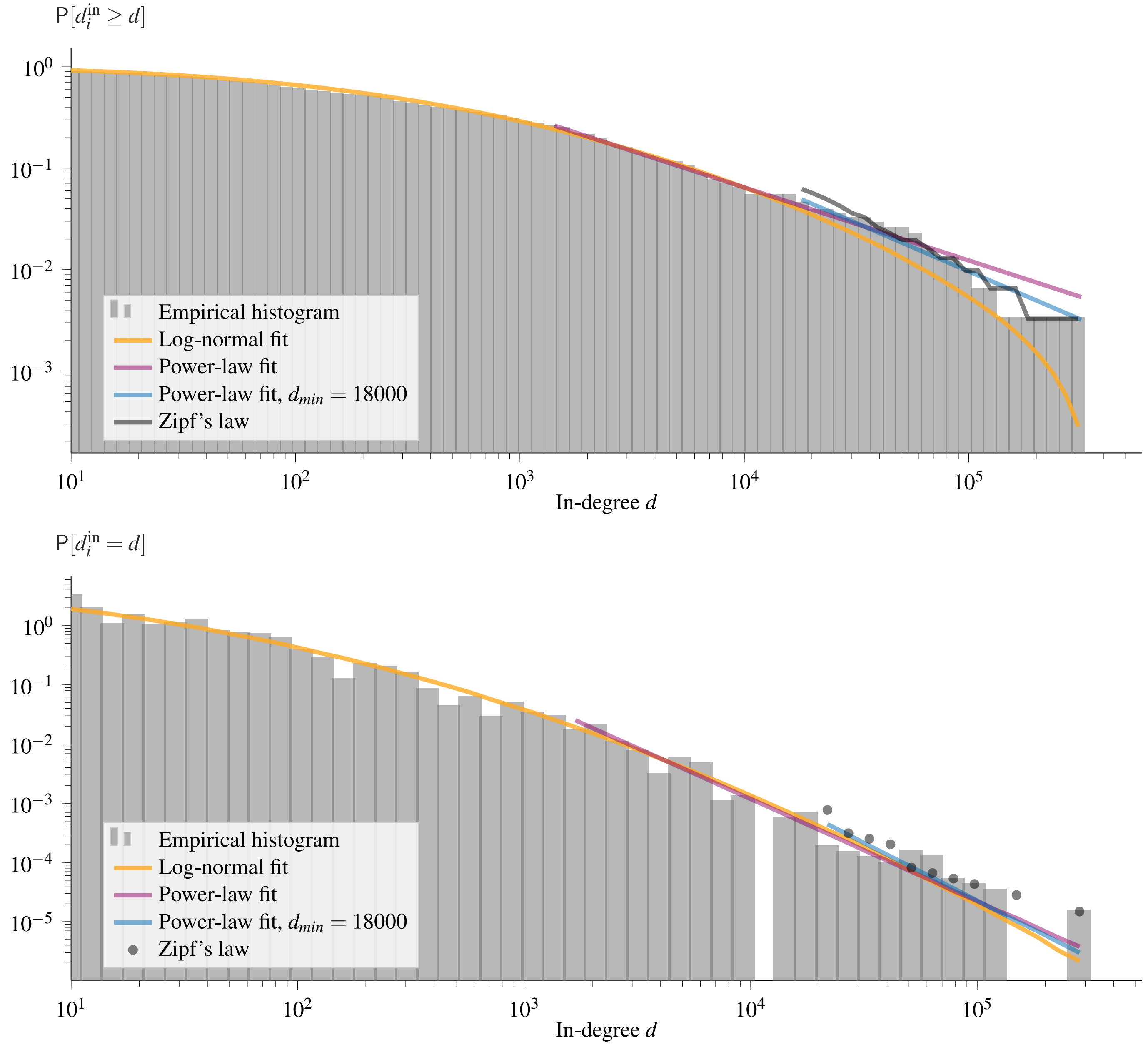

Supplementary Figure 12. Comparison between empirical distribution of the in-degree probability distribution function of the chess data-set and log-normal, power-law, and Zipf's law fit. On top, the Complementary Cumulative distribution function, while on bottom the probability density function are displayed. The log-normal fit has parameters $\mu = 5.57, \sigma = 2.40$. The power-law fit (in purple) has parameters $\alpha = 1.72, \sigma = 0.08, d_{min} = 1425$. The power-law fit with $d_{min} = 18000$ (in blue) has parameters $\alpha = 1.95, \sigma = 0.25$. Finally, the Zipf's law distribution is a computationally tractable approximation of our theoretical distribution, as shown in Supplementary fig. 13.

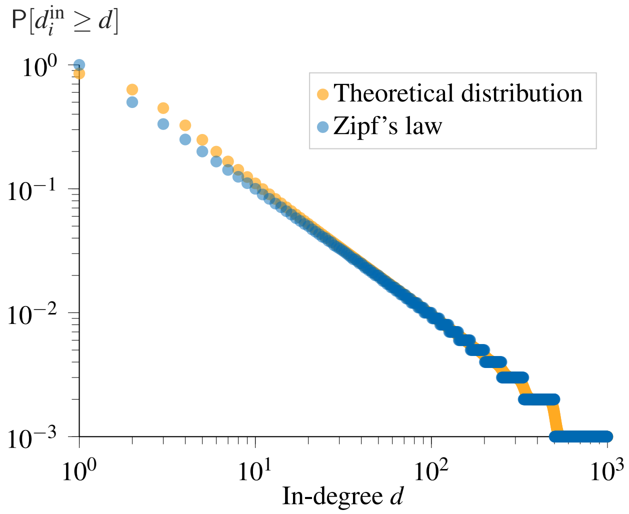

Supplementary Figure 13. Comparison of the Complementary Cumulative distribution functions of our theoretical distribution (with uniform probability in the meeting process) and a pure Zipf's law, for a network with $N = 1000$ nodes.

Followers' out-degree distribution

As mentioned in the manuscript, the followers' out-degree distribution found in our Twitch data-sets partly depends on the followee. Supplementary fig. 15 shows the stacked frequencies of the out-degree of the followers of each of the 15 most followed nodes in the two data-sets. In particular, we observe that the top nodes have a larger percentage of followers of out-degree $d^{out} = 1$. In other words, the top nodes are more likely to be followed by users that do not follow any other node in the category of interest. We conjecture that this is the effect of the recommendation systems behind the Twitch platform. On the other hand, a large audience with out-degree 1 can also be due to a non-perfect alignment of the node's interest, as for example for the node ranked 11 and 13 in the poker data-set (see also the audience overlap index in Supplementary fig. 14). This can be the case of a node that accumulated followers from another category and recently switched to playing poker. Thus, its audience is related to another category.

Ultimately, we believe that further investigation is needed on the out-degree distribution. While our work is currently limited by the sampling bias, we also believe that the model can be enriched to capture, e.g., heavier tails compared to those predicted by our simil-exponential distribution. For example, it is possible to keep the meritocratic principle, but "relaxing" the dynamics by using the average received quality instead of the maximum quality as a threshold for a new followee. This would naturally lead to a larger spectrum of out-degree.

Audience overlap analysis

Finally, for completeness, we present the results on the audience overlap index for the poker data-set (see Supplementary fig. 14). Compared to the simulations results in fig. 10a in the manuscript, here the nodes tend to share a slightly larger audience. Again, that could be the result of a recommendation system that suggests users to follow nodes similar to those they already follow.

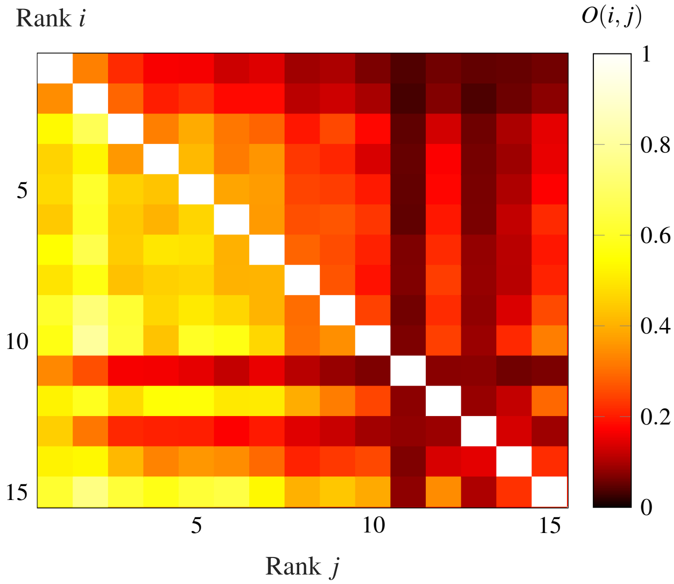

Supplementary Figure 14. Followers' overlap results among the top 15 nodes in the poker data-set.

Supplementary References

1. Barabási, A.-L. & Albert, R. Emergence of scaling in random networks. *science* **286**, 509–512 (1999).
2. Teng, S.-Y., Yeh, M.-Y. & Chuang, K.-T. Toward understanding the mobile social properties: An analysis on instagram photo-sharing network. In *2015 IEEE/ACM International Conference on Advances in Social Networks Analysis and Mining (ASONAM)*, 266–269 (IEEE, 2015).
3. Hosseinmardi, H. *et al.* Detection of cyberbullying incidents on the instagram social network. *arXiv preprint arXiv:1503.03909* (2015).
4. Wattenhofer, M., Wattenhofer, R. & Zhu, Z. The youtube social network. In *Sixth international AAAI conference on weblogs and social media* (2012).
5. https://twitchadvertising.tv/audience/.
6. https://dev.twitch.tv/docs/api/reference.
7. D L. King, J. B., P. H. Del Fabbro & Potenza, M. N. *Problematic online gaming and the COVID-19 pandemic* (Journal of Behavioral Addictions 9(2):184-186, 2020).
8. https://sullygnome.com/game/Poker/longtermstats.
9. https://sullygnome.com/game/Chess.

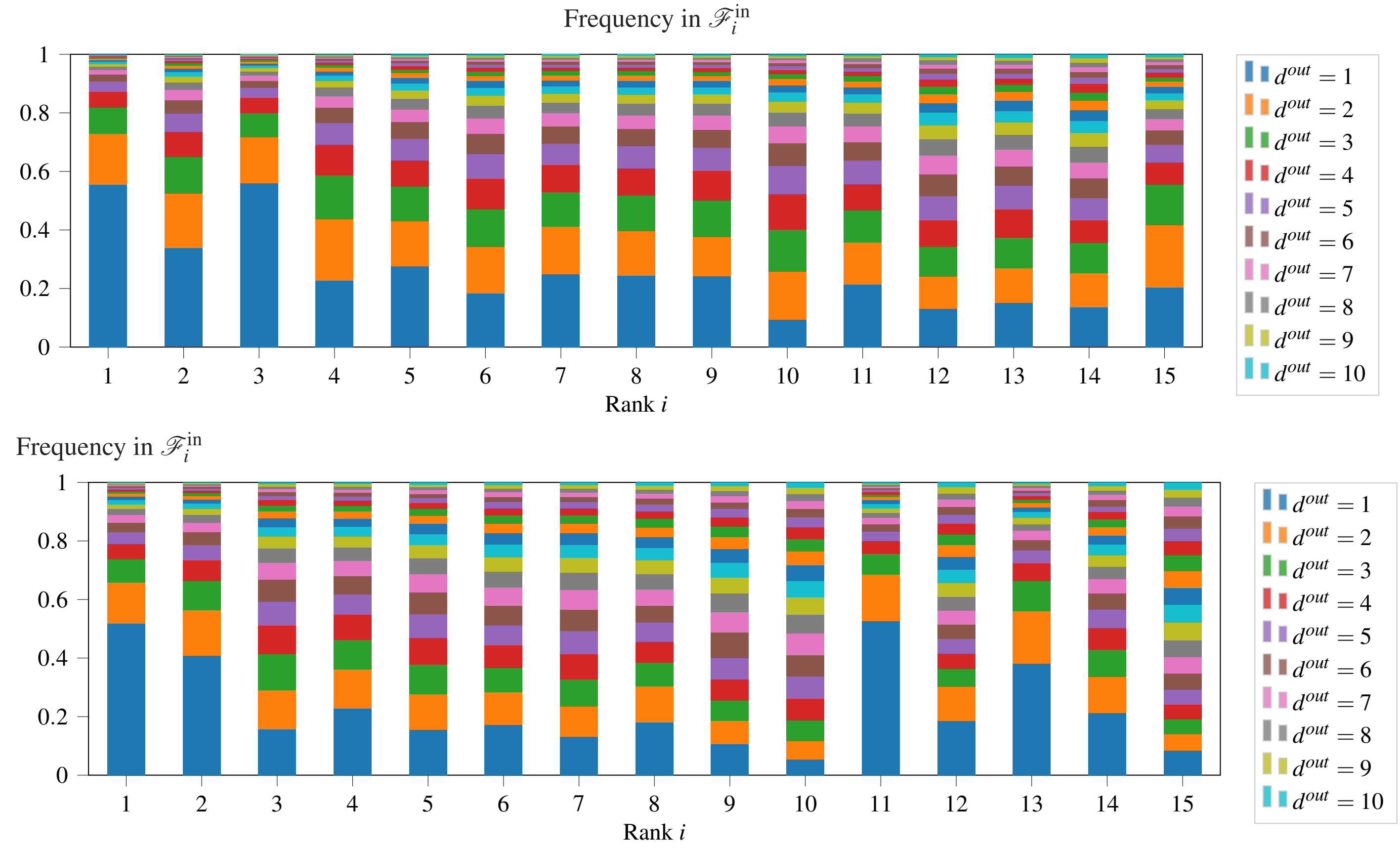

Supplementary Figure 15. The stacked histograms show, for each of the top 15 users in the two data-sets (chess on top, poker on bottom), the followers' out-degree distribution. In other words, we partition the set of followers of each of the top 15 nodes by their out-degree.
